# LKAN: A Kolmogorov–Arnold Network-Based Framework with Long-History Statistical Regularization for IMU Trajectory Estimation

**DOI:** 10.3390/s26123649

**Published:** 2026-06-08

**Authors:** Wenhao Wang, Yanping Zhu, Yixuan Tang, Chengjin Hong

**Affiliations:** 1 School of Wang Zheng Microelectronics, Changzhou University, Changzhou 213159, China; s24060809026@smail.cczu.edu.cn (W.W.); s23060809037@smail.cczu.edu.cn (Y.T.); 2 School of Computer Science and Artificial Intelligence, Changzhou University, Changzhou 213159, China; s24060854021@smail.cczu.edu.cn

**Keywords:** inertial measurement unit (IMU), Kolmogorov–Arnold network (KAN), trajectory estimation, long-range temporal dependencies, statistical regularization, pedestrian dead reckoning (PDR), indoor positioning

## Abstract

High-precision indoor trajectory estimation using pure Inertial Measurement Units (IMUs) remains challenging due to severe cumulative drift and the complexity of modeling nonlinear dynamics. This paper proposes LKAN, a novel end-to-end framework that integrates the Kolmogorov–Arnold Network (KAN) with Long-History Statistical Regularization (LHSR). We design the KANmer encoder, which fuses Multi-Head Self-Attention with KAN to explicitly capture long-range temporal dependencies and intricate nonlinear features from IMU data. To enhance model robustness, a training-only Long-History Statistical Regularization mechanism is introduced; it effectively suppresses feature distribution drift by enforcing historical statistical consistency. Extensive evaluations on three public datasets demonstrate that LKAN significantly outperforms state-of-the-art methods in IMU-only pedestrian localization. Specifically, on the iIMU-TD dataset, LKAN achieves an Absolute Trajectory Error (ATE) of 2.04 m and a Relative Trajectory Error (RTE) of 2.72 m, representing a reduction of 33.8% and 31.1%, respectively, compared to the second-best ResT-IMU. Results on the RoNIN dataset further validate the superiority of LKAN. These findings confirm that LKAN effectively mitigates error accumulation, providing a reliable, high-precision solution for real-time IMU-based positioning in complex indoor environments.

## 1. Introduction

With the rapid proliferation of the Internet of Things (IoT), intelligent manufacturing, and autonomous systems, high-precision positioning and trajectory estimation have become foundational for applications such as smart warehousing, indoor navigation, augmented reality (AR), and human–computer interaction [1,2]. While the Global Navigation Satellite System (GNSS) provides reliable meter-level or even decimeter-level localization in open outdoor environments [3], its performance severely degrades in "GNSS-denied" areas—such as urban canyons, tunnels, and indoor facilities—due to signal attenuation and multipath interference [4]. Consequently, developing robust indoor positioning technologies is imperative to achieve seamless, ubiquitous localization services.

Existing indoor positioning modalities primarily include Visual Odometry (VO) [5] and wireless signal-based methods (e.g., WiFi, Bluetooth, UWB) [6,7,8]. Although visual methods offer high precision in texture-rich environments, they are susceptible to illumination variations and dynamic occlusions, while their high computational overhead limits deployment on resource-constrained wearable devices. Wireless methods, conversely, suffer from signal fading and environmental instability, hindering consistent reliability. In contrast, Inertial Measurement Units offer a self-contained solution characterized by high sampling rates, low power consumption, and immunity to external environmental interference [9]. However, the inherent sensor noise and stochastic biases in IMUs lead to rapid error accumulation during the integration of acceleration and angular velocity, resulting in significant trajectory drift [10]. Thus, achieving long-term, high-precision pedestrian trajectory estimation using only IMU data remains a formidable challenge.

To mitigate a drift in IMU-only Inertial Odometry (IO), early research focused on classical filtering and Pedestrian Dead Reckoning (PDR) [11,12,13]. While computationally efficient, these heuristic models struggle to characterize the complex, nonlinear, and idiosyncratic nature of human motion. The emergence of deep learning has shifted the paradigm toward data-driven approaches. For instance, IONet utilized Long Short-Term Memory (LSTM) networks to regress displacements by modeling temporal dependencies [14,15]. Nevertheless, recurrent architectures are often plagued by gradient vanishing and error propagation in long sequences [16]. To enhance stability, the RoNIN framework [17,18] employed 1D Temporal Convolutional Networks (TCNs) with residual structures to extract local inertial features. While TCNs offer superior generalization, their fixed receptive fields limit their capacity to capture multiscale nonlinear motion evolutions [19].

More recently, Transformers have been introduced to IO tasks, leveraging self-attention mechanisms for long-range dependency modeling [20,21]. Despite their success in global temporal aggregation, standard Transformers rely on Multi-Layer Perceptrons (MLPs) for feature transformation. MLPs, however, exhibit limited efficacy in explicitly modeling the highly nonlinear, high-frequency dynamics inherent in IMU signals. The Kolmogorov–Arnold Network (KAN) [22] has recently emerged as a promising alternative, offering superior nonlinear fitting capabilities and mathematical interpretability by replacing fixed activation functions on nodes with learnable univariate functions on edges. Although early attempts like CKANIO [23] used KAN-based residual structures to improve accuracy, they primarily functioned as static mapping modules, failing to integrate the essential temporal modeling required for sequential trajectory estimation.

Furthermore, long-term cumulative error remains the primary bottleneck. While strategies like future-conditioned prediction [24,25] show promise, they often introduce non-causal information leakage, making them impractical for real-time inference. To address these challenges-specifically: (1) the difficulty of modeling complex nonlinear dynamics in IMU time-series; (2) the uncertainty of motion evolution; and (3) stability degradation due to noise accumulation—this paper proposes LKAN, a novel robust trajectory estimation framework. LKAN leverages KAN with B-spline-based learnable activation functions to achieve refined modeling of nonlinear IMU dynamics. The main contributions are summarized as follows:

- Development of the KANmer encoder: We propose a hybrid architecture that synergistically integrates KAN with Multi-Head Self-Attention (MHSA). This design combines the global temporal modeling strengths of Transformers with KAN’s superior function approximation for high-frequency nonlinearities, significantly enhancing the representation accuracy of diverse human motion patterns.
- Introduction of Long-History Statistical Regularization (LHSR): To combat feature distribution drift, we design a training-only auxiliary constraint strategy. By mining statistical invariants from historical sequences, LHSR enforces consistency between current latent representations and historical motion distributions. This approach provides a "soft constraint" that enhances global robustness without violating causal constraints or increasing inference latency.
- Comprehensive Validation: Extensive experiments on three benchmark datasets demonstrate that LKAN achieves state-of-the-art performance in both Absolute Trajectory Error (ATE) and Relative Trajectory Error (RTE), validating its robustness and generalization across various complex indoor scenarios.

## 2. Materials and Methods

**Figure 1:**
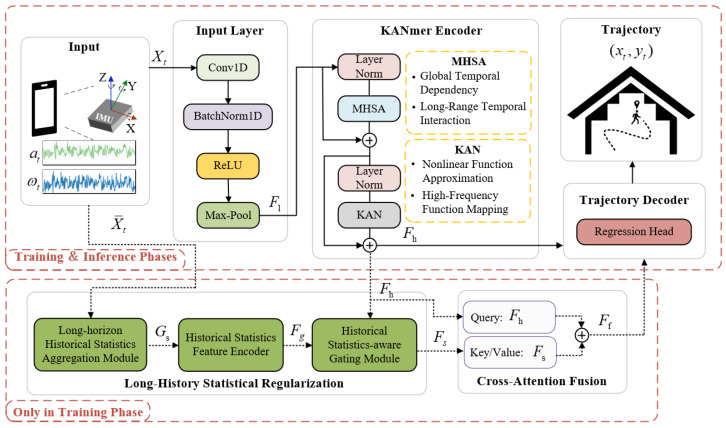
Overall network architecture of the proposed LKAN model for IMU-based indoor pedestrian trajectory estimation. The upper part of the network—including the input layer, KANmer encoder (which integrates Multi-Head Self-Attention with the Kolmogorov–Arnold Network), and the trajectory decoder—is active in both training and inference phases. The lower part, comprising the Long-History Statistical Regularization module and the cross-attention fusion unit, is only engaged during training to extract historical motion statistical constraints and enhance the robustness of the learned features.

As illustrated in Figure 1, the proposed LKAN model takes 3-axis acceleration $a_{t}\in R^{3}$ and angular velocity $\omega_{t}\in R^{3}$ measured by the IMU as inputs.

The raw inertial signals are synchronously divided into two sliding windows of different lengths and fed into two dedicated functional branches.

Main inference branch (active in both training and inference phases): The input layer preprocesses and samples the short-window IMU sequence $X_{t}\in R^{32\times {\overset{˘}{L}}_{t}\times 6}$, where ${\overset{˘}{L}}_{t}$ denotes the adaptive window length at time step *t* after smoothing. The sequence is then passed to the KANmer encoder. This encoder combines Multi-Head Self-Attention with the Kolmogorov–Arnold Network to capture long-range temporal dependencies and model high-frequency nonlinear dynamics, producing the robust historical trajectory feature $F_{h}\in R^{32\times 64\times 64}$.

Training-only regularization branch (active exclusively during training): The long-horizon historical IMU sequence ${\bar{X}}_{t}\in R^{32\times L_{h}\times 6}$, where $L_{h}$ denotes the length of the long-horizon historical observation window, is processed by the Long-History Statistical Regularization module. This module performs statistical aggregation over long-term historical observations to extract motion statistical invariants, yielding the historical motion constraint feature $F_{s}\in R^{32\times 64\times 64}$.

During training, $F_{s}$ is integrated with $F_{h}$ via a cross-attention module to enhance the robustness of the trajectory feature representation. The fused features are then fed into the trajectory decoder for coordinate regression. During inference, the LHSR branch is deactivated, and only $F_{h}$ from the KANmer encoder is passed directly to the decoder, which produces accurate 2D pedestrian trajectory estimations.

### 2.1. Adaptive Input Window and Base Feature Extraction

To enhance the model’s responsiveness to highly dynamic motion scenarios, an adaptive sliding window module based on the Euclidean norm of the linear acceleration $∥a_{t}{∥}_{2}$ and angular velocity $∥\omega_{t}{∥}_{2}$ is added at the front end of the encoder. This module dynamically adjusts the input sequence length by modifying the temporal receptive field in real time. The core logic is as follows: when $∥a_{t}{∥}_{2}$ or $∥\omega_{t}{∥}_{2}$ is large (corresponding to transient motions such as acceleration, deceleration, or turning), the window shortens to enhance sensitivity to instantaneous changes; when both are small (during uniform steady motion), the window expands to integrate steady-state contextual information.

The window length is defined as

(1) $$L_{t}=\mathrm{clip}\left(L_{0}-\alpha_{a}∥a_{t}{∥}_{2}-\alpha_{\omega }{∥\omega_{t}∥}_{2},L_{\min},L_{\max}\right),$$

where $L_{0}$ denotes a baseline window length, and $\alpha_{a}$ and $\alpha_{\omega }$ control the sensitivity of the window to variations in acceleration and angular velocity, respectively. The clip(·) function is defined as

(2) $$\mathrm{clip}\left(x,L_{\min},L_{\max}\right)=\min\left(\max\left(x,L_{\min}\right),L_{\max}\right),$$

which constrains the window length within the predefined range $\left[L_{\min},L_{\max}\right]$. This prevents instability caused by excessively short windows or delayed responses due to overly long ones.

To suppress drastic fluctuations of the window between consecutive frames, exponential smoothing is further applied:

(3) $${\overset{˘}{L}}_{t}=\beta {\overset{˘}{L}}_{t-1}+\left(1-\beta \right)L_{t},$$

where β=0.8 is the smoothing coefficient. This parameter selection is based on empirical observation and dataset statistics, consistent with common practice in IMU-based adaptive segmentation [26]. It ensures that the sliding window is short enough to be responsive to transient motions, such as acceleration, deceleration, or turning, while long enough to integrate steady-state contextual information during uniform motion.

The IMU sequence output by the windowing mechanism is then fed into the base feature extraction layer for preliminary representation. A 1D convolution (kernel = 7, stride = 2) expands 6-dimensional raw IMU signals into a 64-dimensional feature space. Batch normalization stabilizes the training process, followed by a ReLU activation function to introduce nonlinearity. Max pooling compresses the temporal dimension while preserving key responses. After the above processing, structured and discriminative low-level features $F_{l}\in R^{32\times 64\times 64}$ are obtained, which are then passed to the subsequent KANmer encoder for deep feature extraction.

### 2.2. KANmer Encoder

**Figure 2:**
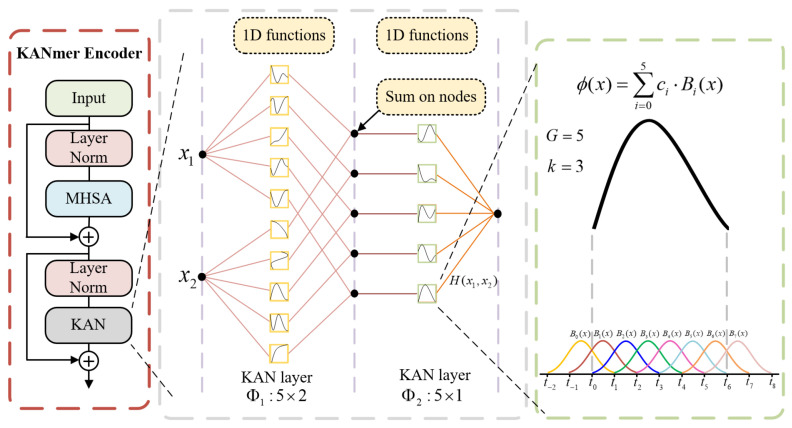
Architecture of the proposed KANmer encoder. The encoder integrates Multi-Head Self-Attention with a two-layer Kolmogorov–Arnold Network to capture long-range temporal dependencies and nonlinear inertial dynamics. The KAN module adopts a layer-wise expansion structure, where learnable univariate transformations are first applied to the inputs $x_{1}$ and $x_{2}$, followed by summation-based aggregation to produce the output $H(x_{1},x_{2})$. The notation (5×2) denotes five intermediate univariate function outputs generated from two input variables, while (5×1) represents their aggregation into the final output. The right panel illustrates the B-spline-based learnable univariate functions ϕ(x), where *G* and *k* denote the grid size and spline order, respectively. In this work, cubic B-splines (k=3) with grid size G=5 are adopted, resulting in G+k=8 basis functions.

The KANmer encoder proposed in this paper is the core module of the LKAN model. It consists of a Multi-Head Self-Attention module and a KAN connected sequentially [27], as shown in Figure 2. This architecture is explicitly designed to align with the overall modeling philosophy illustrated in the figure, where long-range temporal dependency extraction is first performed, followed by dimension-wise nonlinear transformation. The motivation is to jointly model global temporal dynamics and complex nonlinear inertial mappings in IMU-based trajectory estimation. Specifically, MHSA is responsible for capturing long-range temporal dependencies and global contextual information from IMU sequences, while the KAN module performs subsequent nonlinear feature transformation in a dimension-wise manner. This decoupled design enables a structured representation that separates temporal modeling from nonlinear inertial function approximation.

#### 2.2.1. Multi-Head Self-Attention Mechanism

To effectively model the multi-scale temporal dependencies of IMU sequences, the Multi-Head Self-Attention mechanism is employed. Its core attention calculation is defined as

(4) $$\mathrm{Attention}\left(Q,K,V\right)=\mathrm{softmax}\left(\frac{QK^{⊤}}{\sqrt{d_{k}}}\right)V,$$

where *Q*, *K*, and *V* denote the query, key, and value matrices, respectively, and $d_{k}$ is the dimensionality of the key vectors. The softmax function is defined as

(5) $$\mathrm{softmax}\left(z_{i}\right)=\frac{\exp(z_{i})}{\sum_{j}\exp\left(z_{j}\right)},$$

More details can be found in [20]. The Multi-Head Self-Attention mechanism computes attention over multiple feature subspaces in parallel, enabling the model to capture both short-term variations and long-range temporal dependencies in IMU signals. This temporally enriched representation provides a global contextual encoding of motion dynamics, which serves as a structured input for subsequent nonlinear modeling in the KAN module.

#### 2.2.2. KAN Layer

The output feature $F_{m}\in R^{32\times 64\times 64}$ obtained from the MHSA module is further processed by the KAN layer to model complex nonlinear dependencies in IMU signals. As a structured alternative to conventional MLPs, KAN replaces fixed activation functions with learnable univariate functions on each connection, enabling more expressive and interpretable nonlinear function approximation for motion dynamics.

A KAN consisting of *K* layers can be formulated as a nested function composition:

(6) $$\mathrm{KAN}\left(Z\right)=\left(\Phi_{K-1}\circ \Phi_{K-2}\circ \cdots \circ \Phi_{0}\right)\left(Z\right),$$

In this work, we consider the two-layer case (K=2), which is sufficient to model the nonlinear mapping while maintaining computational efficiency.

For each layer $\Phi_{k}$, the mapping from input dimension *j* to output dimension *i* is defined by a set of learnable univariate functions:

(7) $$Z_{k+1,i}=\sum_{j=1}^{n_{\mathrm{in}}}\phi_{k,i,j}\left(Z_{k,j}\right),\;i=1,\ldots ,n_{\mathrm{out}},$$

Each function $\phi_{k,i,j}\left(\cdot \right)$ is parameterized using a B-spline expansion:

(8) $$\phi \left(x\right)=\sum_{i=0}^{5}c_{i}\cdot B_{i}\left(x\right),$$

where $c_{i}$ are learnable coefficients and $B_{i}\left(x\right)$ denotes B-spline basis functions [28].

In IMU-based trajectory estimation, this decomposition is particularly important, as human motion exhibits strong nonlinearities arising from gait variability, posture transitions, and directional changes. By assigning independent learnable functions to each inertial dimension, KAN decomposes high-dimensional motion mappings into a set of interpretable and adaptive nonlinear transformations, improving both modeling flexibility and robustness.

From an interpretability perspective, the KANmer encoder provides a traceable representation of the trajectory estimation process. The explicit functional formulation of KAN enables analysis of the contribution of each inertial dimension through learned spline coefficients, while MHSA attention weights reflect the temporal importance of different motion segments. This dual interpretability allows fine-grained analysis of motion dynamics and facilitates error source identification in trajectory estimation.

Overall, the proposed KANmer encoder explicitly decouples temporal dependency modeling and nonlinear inertial mapping into two sequential stages. This differs from conventional Transformer-based architectures, where nonlinear transformations are implicitly embedded within feed-forward networks without explicit functional decomposition. By integrating MHSA and KAN in a structured manner, the model achieves both expressive motion representation and interpretable decomposition of inertial contributions, leading to improved robustness under complex and non-stationary motion conditions.

### 2.3. Long-History Statistical Regularization Module

**Figure 3:**
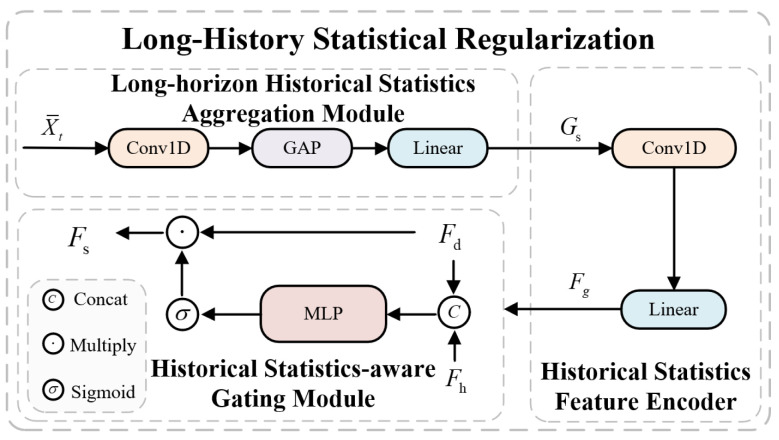
Overall structure of the Long-History Statistical Regularization module. The core idea of the proposed module is to leverage the statistical stability of observed historical data to construct global constraints. A history-aware statistical gating mechanism is introduced to adaptively select informative constraint signals, enabling statistical refinement of historical trajectory features rather than providing independent predictions. This design achieves a balance between enforcing long-term statistical consistency and preserving the modeling capacity for local instantaneous motion dynamics.

To address the issues of error accumulation and feature distribution drift caused by local integration in IMU-based trajectory estimation, this paper proposes a Long-History Statistical Regularization module. This module is designed as a training-only regularization strategy, which constructs statistical priors of motion patterns by mining invariant properties from previously observed IMU sequences. It imposes a soft constraint that encourages the feature representation at the current time step to remain consistent with historical motion distributions. In this way, the proposed method effectively suppresses global drift in trajectory estimation while strictly adhering to the causality principle. Notably, the LHSR module is only activated during training and introduces no additional computational overhead during inference, thus preserving the model’s real-time deployment capability. The overall architecture is illustrated in Figure 3, and the detailed process is described as follows.

- Long-horizon History Statistical Aggregation;

Given the input IMU sequence ${\bar{X}}_{t}$, where $L_{h}$ denotes the length of the observed history, local temporal features are first extracted via a 1D convolution. Subsequently, global average pooling (GAP) is applied along the temporal dimension to aggregate long-term motion statistics. This operation effectively acts as a low-pass filter, enabling the extracted representation to capture stable statistical properties while suppressing high-frequency instantaneous variations. A linear projection is then applied to obtain the historical statistical feature:

(9) $$G_{s}=\mathrm{Linear}\left(\mathrm{GAP}(\mathrm{Conv}1D(X))\right),$$

where $\mathrm{Conv}1D{\left(X\right)}_{t}=\sum_{k}W_{k}X_{t-k}+b$ and Linear(x)=Wx+b. Here, $W_{k}$ and *b* denote the convolution kernel weights and bias, respectively, while *W* and *b* represent the weight matrix and bias vector of the linear transformation. The resulting feature $G_{s}\in R^{32\times 64}$ captures the statistical characteristics of the observed IMU history.

- Historical Statistical Feature Encoding

To further enhance representational capacity, the aggregated statistical feature is passed through a lightweight encoder composed of a 1D convolution and a linear layer. This yields a high-dimensional statistical embedding:

(10) $$F_{g}=\mathrm{Linear}\left(\mathrm{Conv}1D(G_{s})\right),$$

where $F_{g}\in R^{32\times 64\times 64}$ shares the same dimensionality as the historical trajectory feature $F_{h}$, facilitating subsequent interaction and fusion. Importantly, $F_{g}$ is derived solely from historical IMU observations and serves as a statistical regularization signal.

- Statistics-Aware Gating Mechanism

Considering that the extracted long-term statistics may contain noise or components inconsistent with the current motion context, a statistics-aware gating mechanism is introduced to adaptively filter reliable constraint information.

Let $F_{h}$ denote the historical trajectory feature produced by the KANmer encoder. The discrepancy between statistical priors and local features is first computed as

(11) $$F_{d}=F_{g}-F_{h}.$$

Next, the two features are concatenated and passed through a multi-layer perceptron followed by a Sigmoid activation to generate a gating weight:

(12) $$W=\sigma \left(\mathrm{MLP}(\left[F_{h};F_{g}\right])\right),$$

where [;] denotes channel-wise concatenation and W∈[0,1] reflects the reliability of the statistical constraint under the current context.

The final filtered statistical constraint is obtained via element-wise modulation:

(13) $$F_{s}=W⊙F_{d},$$

where ⊙ denotes element-wise multiplication. This mechanism amplifies stable statistical patterns (W→1) while suppressing unreliable or noisy components (W→0), enabling the model to refine feature representations without introducing independent prediction bias.

- Feature Fusion and Trajectory Prediction

The refined statistical constraint $F_{s}$ is integrated with the historical feature $F_{h}$ via a cross-attention mechanism. Specifically, $F_{h}$ serves as the query, while the statistical constraint is used as the key and value. Through attention-based interaction, the model incorporates global statistical consistency into local temporal features, enhancing robustness against drift while preserving temporal continuity.

The fused representation $F_{f}\in R^{32\times 64\times 64}$ is then fed into the decoder to produce the final trajectory prediction ${\hat{T}}_{t}$.

### 2.4. Loss Function

To ensure accurate trajectory estimation while enforcing long-history statistical consistency, a joint loss function is constructed by integrating the basic prediction loss with the statistical regularization loss. Using mean squared error (MSE) as the core metric, the proposed objective enables collaborative optimization of local accuracy and global consistency. Notably, all loss terms are computed based solely on observed historical data, strictly adhering to the causality constraint.

#### 2.4.1. Basic Prediction Loss

The basic prediction loss $L_{1}$ measures the discrepancy between predicted velocity and ground-truth velocity, providing fundamental supervision for trajectory estimation and enabling accurate modeling of local instantaneous motion dynamics. It is defined as

(14) $$L_{1}=\frac{1}{n}\sum_{i=1}^{n}{\left({\hat{v}}_{i}-v_{i}\right)}^{2},$$

where *n* denotes the number of time steps within a trajectory sequence, $v_{i}$ is the ground-truth velocity at the *i*-th time step, ${\hat{v}}_{i}$ is the corresponding prediction, and *i* indexes time steps within each trajectory window.

This loss is formulated as a mean squared error over the entire trajectory sequence, rather than supervision at a single time instant. Minimizing $L_{1}$ enforces time-step-wise regression consistency across the full sequence and reduces instantaneous estimation errors.

#### 2.4.2. Long-History Statistical Regularization Loss

To mitigate error accumulation caused by local inertial integration, a Long-History Statistical Regularization loss is introduced. Instead of directly supervising trajectory predictions, this term enforces consistency between statistical representations derived from historical features and statistical characteristics extracted from ground-truth trajectory sequences. In this way, global motion consistency is imposed at the representation level.

The overall training objective is formulated as

(15) $$L=L_{1}+\gamma L_{2},$$

where γ∈[0,1] is a balancing coefficient controlling the strength of the statistical regularization term. $L_{2}$ is defined as

(16) $$L_{2}=\frac{1}{n}\sum_{i=1}^{n}{\left({\hat{v}}_{h,i}-v_{s,i}\right)}^{2},$$

where *n* denotes the number of time steps in the trajectory window, and the loss is computed as the mean squared error over the entire sequence, ${\hat{v}}_{h,i}$ denotes the representation derived from the learned historical statistical features, and $v_{s,i}$ represents statistical velocity features extracted from ground-truth trajectory sequences using a temporal-window-based aggregation operator.

Both $L_{1}$ and $L_{2}$ are computed over aligned temporal windows, ensuring consistent optimization across prediction and statistical alignment objectives.

Unlike conventional auxiliary losses, $L_{2}$ does not introduce an additional prediction branch. Instead, it serves as a feature-level constraint that encourages the learned representations to align with stable long-term motion statistics, thereby improving temporal coherence of the learned model.

From an optimization perspective, this statistical regularization acts as a distribution-level constraint that helps reduce the effects of bias induced by local inertial integration, leading to improved long-term stability in trajectory estimation.

### 2.5. Computational Complexity Analysis

This section presents a theoretical analysis of the computational complexity of the proposed LKAN model and further validates its efficiency through measured inference time. On an NVIDIA GTX 3060 Ti GPU, the average inference time per sequence is approximately 6.95 ms, demonstrating that the proposed model satisfies the real-time requirements of trajectory estimation tasks. Since the LHSR module is only introduced as a regularization term during training, the primary computational cost during inference is mainly attributed to the KANmer encoder and the decoder. The inference-stage and training-stage complexities are analyzed separately below.

#### 2.5.1. Inference Complexity

During inference, the main computational components of the model include the Multi-Head Self-Attention mechanism and the KANmer encoder based on the Kolmogorov–Arnold Network.

First, for the MHSA module, let the input sequence length be *L*, feature dimension be *D*, and number of attention heads be *H*. The single-head self-attention requires constructing an attention matrix with pairwise token interactions, resulting in a complexity of $O(L^{2}D)$. Therefore, the multi-head version scales to $O(HL^{2}D)$.

Second, for the standard Transformer MLP, let the input dimension be $n_{in}$ and output dimension be $n_{out}$. The computation is mainly dominated by linear projections, giving a complexity of $O(n_{in}\cdot n_{out})$.

In contrast, the proposed KAN module replaces fixed activation functions with learnable B-spline-based nonlinear mappings. Let *K* denote the network depth, and let *C* denote the number of control points in each B-spline activation function. Since each univariate spline is parameterized by *C* control points, a single KAN layer has a computational complexity of $O(n_{in}\cdot n_{out}\cdot C)$. Accordingly, a *K*-layer KAN has a complexity of $O(K\cdot n_{in}\cdot n_{out}\cdot C$). In B-spline-based KAN implementations, the number of control points is typically defined as C=G+k, where G denotes the grid size and k denotes the spline order. In this work, we set K=2, G=5, and k=3, ensuring that the additional computational overhead introduced by KAN remains manageable while significantly improving the nonlinear representation capability.

The decoder consists of fully connected layers and a lightweight temporal mapping module. Let the output dimension be $D_{out}$, then its complexity is $O(L\cdot D\cdot D_{out})$. Therefore, the overall inference complexity of the LKAN model can be expressed as

(17) $$O(HL^{2}D+K\cdot n_{in}\cdot n_{out}\cdot C+L\cdot D\cdot D_{out})$$

Since *D* and $D_{out}$ are typically constant-scale hyperparameters, the overall complexity is mainly dominated by the MHSA term $O(L^{2})$, consistent with standard Transformer-based temporal models.

#### 2.5.2. Training Complexity

During training, the model additionally introduces a Long-Horizon Statistical Consistency Regularization module to constrain long-term motion statistics and improve global trajectory consistency. This module is only activated during training and does not participate in inference-time forward propagation.

Specifically, the LHSR module consists of a 1D convolution and a global average pooling operation. Let the input channel dimension be $C_{in}$, output channel dimension be $C_{out}$, kernel size be *k*, and sequence length be *L*. The convolution operation has complexity $O(L\cdot C_{in}\cdot C_{out}\cdot k)$, while global average pooling contributes $O(L\cdot C_{out})$.

Thus, the overall training complexity of LHSR is

(18) $$O(L\cdot C_{in}\cdot C_{out}\cdot k+L\cdot C_{out})$$

Since LHSR is an auxiliary training-only branch, it does not affect inference-time computation. Therefore, the proposed model improves long-sequence trajectory estimation stability without sacrificing real-time performance.

## 3. Experiments and Results

To comprehensively evaluate the localization accuracy, robustness, and effectiveness of the proposed LKAN model for IMU-only trajectory estimation, extensive experiments are conducted on several widely used public benchmarks. The evaluation includes ablation studies, module configuration analysis, mechanism validation, and quantitative comparisons with state-of-the-art methods. In addition, qualitative trajectory visualization is provided to assess fitting performance, and the influence of key hyperparameters is systematically analyzed. All experiments follow standard evaluation protocols in inertial navigation, ensuring a fair and comprehensive validation of the proposed method.

### 3.1. Experimental Setup

All experiments were implemented using PyTorch 2.0.0 with Python 3.10. The hardware platform consisted of an NVIDIA GTX 3060 Ti GPU (NVIDIA Corporation, Santa Clara, CA, USA), an Intel Core i7-12700H CPU (Intel Corporation, Santa Clara, CA, USA), and 32 GB RAM (Kingston Technology Corporation, Fountain Valley, CA, USA). The Adam optimizer was employed with an initial learning rate of $1\times 10^{-4}$, along with an adaptive decay strategy. The weight decay was set to $1\times 10^{-1}$, the dropout rate was 0.1, the batch size was 32, and the model was trained for 500 epochs.

### 3.2. Experimental Evaluation Metrics

Two standard metrics in inertial navigation, Absolute Trajectory Error (ATE) and Relative Trajectory Error (RTE), were adopted to evaluate model performance. Both metrics were measured in meters (m), where lower values indicated better accuracy and stability. All results were averaged over multiple runs to reduce randomness.

- Absolute Trajectory Error (ATE);

ATE evaluates the global consistency between the predicted trajectory and the ground truth. The predicted trajectory was first aligned using a similarity transformation. The root mean square error is defined as

(19) $$\mathrm{ATE}=\sqrt{\frac{1}{N}\sum_{i=1}^{N}{∥p_{i}-q_{i}∥}^{2}},$$

where $p_{i}$ and $q_{i}$ denote the aligned predicted and ground-truth positions, respectively, and *N* is the number of samples.

- Relative Trajectory Error (RTE).

RTE measures the local drift over short time intervals, reflecting error accumulation behavior. For a fixed interval Δ, it is defined as

(20) $$\mathrm{RTE}\left(\Delta \right)=\sqrt{\frac{1}{M}\sum_{j=1}^{M}{∥\left(p_{j+\Delta }-p_{j}\right)-\left(q_{j+\Delta }-q_{j}\right)∥}^{2}},$$

where *M* is the number of evaluated segments, and $p_{j}$, $q_{j}$ denote predicted and ground-truth positions. The parameter Δ represents the evaluation horizon. Following standard protocols in inertial navigation and SLAM benchmarks [29], a fixed temporal interval in discrete time steps was adopted. This setting is widely used in relative pose error evaluation to measure short-term trajectory drift and ensure consistent comparison across methods. Given the IMU sampling rate of 200 Hz (0.005 s per frame), we set Δ=20 time steps. This fixed setting was used throughout all experiments to ensure a consistent and reproducible evaluation protocol.

### 3.3. Experimental Datasets

- RoNIN: It is a widely used benchmark containing approximately 42.7 h of multi-device IMU data across 276 sequences, including various carrying modes (e.g., handheld and pocket) and natural human motion patterns [17].
- iIMU-TD: It is a practical dataset with both indoor and outdoor scenarios, comprising 2.41 h of data and a total travel distance of 10.4 km [30].
- OXIOD: It is a cross-scenario dataset featuring complex indoor motion environments, commonly used to evaluate generalization capability [31].

### 3.4. Experimental Results and Analysis

#### 3.4.1. Core Component Ablation Study

To verify the necessity and effectiveness of the three core components in the LKAN model, namely the adaptive window module (AWM), the KANmer encoder, and the Long-History Statistical Regularization (LHSR) module, a controlled ablation study was conducted on the RoNIN and iIMU-TD datasets. Each component was sequentially removed while keeping all other settings unchanged. When the KANmer encoder was ablated, it was replaced with an MLP layer of identical input dimensionality while retaining the Multi-Head Self-Attention module. The evaluation was performed using ATE and RTE, and the results are summarized in Table 1.

The results show that removing the KANmer encoder leads to the most significant performance degradation across both datasets. Specifically, the ATE increases by 11.7% (RoNIN) and 40.2% (iIMU-TD), while the RTE increases by 3.5% and 29.8%, respectively. This demonstrates that the KANmer encoder plays a critical role in modeling nonlinear temporal dependencies by combining the long-range dependency modeling capability of self-attention with the adaptive nonlinear fitting ability of KAN, thereby significantly improving both global consistency and local trajectory coherence.

Removing the LHSR module results in a moderate but consistent performance drop, with ATE increasing by 5.6% (RoNIN) and 21.6% (iIMU-TD), and RTE increasing by 1.6% and 16.5%, respectively. This confirms that relying solely on local temporal modeling leads to cumulative drift, while the proposed statistical regularization effectively suppresses error accumulation by enforcing long-term statistical consistency.

In contrast, removing the AWM causes relatively smaller changes in ATE but noticeable degradation in RTE on the iIMU-TD dataset (+19.1%), indicating its importance in handling diverse motion dynamics. By dynamically adjusting the temporal receptive field according to IMU signal variations, AWM improves robustness under both high-dynamic and smooth motion conditions.

Overall, the ablation results reveal that the KANmer encoder is the primary contributor to performance gains, the LHSR module provides essential global regularization to suppress drift, and the AWM enhances adaptability to varying motion patterns. The three components work in a complementary manner to achieve accurate and stable trajectory estimation.

#### 3.4.2. LHSR Stage Configuration Comparison Experiment and Mechanistic Ablation Experiment

To comprehensively validate the effectiveness of the LHSR module, its behavior under different execution stages, and the necessity of causality constraints, an integrated ablation study was conducted on the RoNIN and iIMU-TD datasets using ATE and RTE as evaluation metrics. With the network architecture and hyperparameters fixed, five configurations were evaluated: (1) LHSR enabled only during training (full long-history window), (2) LHSR enabled only during inference, (3) LHSR enabled during both training and inference, (4) LHSR removed, and (5) a degraded variant where the statistical window is reduced to one-fifth of its original length to destroy long-term motion structure. The experimental results are shown in Table 2.

The results show that enabling LHSR only during training achieves the best trade-off between accuracy and practicality, yielding the lowest errors across both datasets. Compared with the inference-only setting, this configuration reduces ATE by approximately 19% (RoNIN) and 19.7% (iIMU-TD), and RTE by up to 14.0% and 1.8%, demonstrating that statistical constraints must be learned during training to be effective.

In contrast, enabling LHSR only during inference leads to the worst performance, with significant increases in both ATE and RTE. This is because the statistical constraint is not aligned with the learned feature representation, resulting in inconsistency with the prediction outputs.

Although enabling LHSR in both training and inference stages yields slight numerical improvements, it introduces information leakage and violates the causality principle, making it unsuitable for real-world deployment. Therefore, LHSR is applied exclusively during training in the final model.

Furthermore, removing the LHSR module consistently degrades performance across all metrics, while reducing the statistical window leads to intermediate performance. This verifies that the performance gain of LHSR originates from modeling long-history motion statistical structures rather than applying simple statistical constraints. Weakening the long-term aggregation capability directly limits its effectiveness.

#### 3.4.3. Experiment on Complexity and Efficiency Comparison of Different Model Configurations

To analyze the trade-off between computational cost and performance improvement, we compared the parameter size and inference efficiency of different model configurations on the iIMU-TD dataset. Starting from a baseline Transformer model, the KANmer encoder and the LHSR module were progressively introduced while keeping the backbone architecture and training strategy unchanged. The experimental results are shown in Table 3.

The results show that introducing the KANmer encoder brings substantial performance gains with minimal overhead, reducing ATE and RTE by 20.8% and 15.7%, respectively, while increasing the parameter size by only 0.07 M and inference time by 0.31 ms. After further incorporating the LHSR module, the overall parameter and latency increments remain within 0.5% and 8.1%, whereas the performance improvements reach 34.8% (ATE) and 27.7% (RTE) compared to the baseline.

These results demonstrate that the proposed modules achieve significant accuracy improvements at negligible computational cost, leading to an effective balance between efficiency and performance. This validates the practical feasibility of the LKAN model for real-time IMU-only trajectory estimation.

#### 3.4.4. Comparison Experiment with Other Methods

To verify the superiority of the proposed LKAN model, quantitative comparisons were conducted against several state-of-the-art IMU-only trajectory estimation methods, including RoNIN-ResNet, RoNIN-LSTM, RoNIN-TCN [17], ResT-IMU [30], IMUNet [32], and CKANIO. Experiments were performed on the RoNIN, iIMU-TD, and OXIOD datasets using ATE and RTE as evaluation metrics. The experimental results are shown in Table 4.

The results demonstrate that LKAN consistently achieves superior or highly competitive performance across different datasets. On the iIMU-TD dataset, LKAN achieves the best results, reducing ATE and RTE by 33.8% and 31.1%, respectively, compared with the second-best method (ResT-IMU). The improvement is even more significant compared to weaker baselines, reaching over 50% reduction in ATE, which highlights the effectiveness of the proposed design in complex real-world scenarios.

On the RoNIN dataset, LKAN also achieves the best overall performance, with improvements of 6.0% in ATE and 1.9% in RTE compared to the strongest competing method. Although the relative gain is smaller than that on iIMU-TD, the consistent improvement indicates strong generalization under different device configurations and motion patterns.

On the OXIOD dataset, LKAN maintains competitive performance. While its ATE is slightly higher than that of IMUNet, it achieves comparable or better results than most baselines. Notably, the relatively higher RTE compared to RoNIN-TCN suggests that short-term local drift remains challenging in highly complex indoor scenarios, but the overall performance still demonstrates favorable robustness compared with other methods.

Overall, the cross-dataset results confirm that the proposed LKAN model effectively improves trajectory estimation accuracy through the joint design of the KANmer encoder, the LHSR module, and the adaptive window mechanism, achieving strong robustness and generalization across diverse scenarios.

#### 3.4.5. Qualitative Visualization Analysis of Trajectory Estimation

To verify the trajectory fitting performance of the LKAN model more intuitively, this paper conducted two groups of qualitative visualization experiments. The experiments compared the trajectory alignment between LKAN and mainstream methods, and validate the nonlinear feature extraction capability of the KANmer encoder, respectively. Motion sequences with different complexities from the RoNIN and iIMU-TD datasets are selected. The black dashed line represents the ground-truth trajectory, and various solid lines denote the predicted trajectories of different models.

- Multi-Method Trajectory Fitting Comparison Experiment

**Figure 4:**
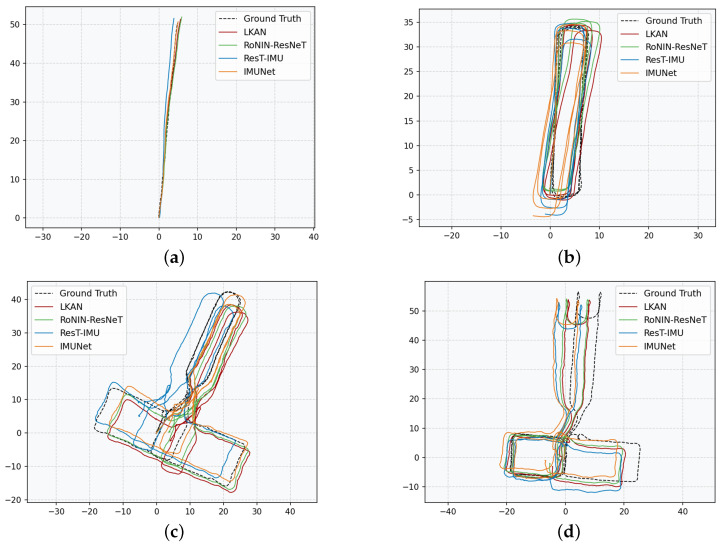
Four representative architectures are considered for comparison, including LKAN (KAN-based), ResT-IMU (Transformer-based), RoNIN-ResNet (ResNet-based), and IMUNet (CNN-based). The qualitative results are visualized on sequences (**a**,**b**) from the iIMU-TD dataset, and (**c**,**d**) from the RoNIN dataset, covering motion patterns ranging from simple linear trajectories to complex high-frequency turning scenarios. Axes denote spatial coordinates in meters (m).

To more intuitively evaluate the trajectory fitting performance of the proposed LKAN model, two groups of qualitative visualization experiments were conducted. These experiments compared trajectory alignment between LKAN and representative mainstream methods, while also validating the nonlinear feature extraction capability of the KANmer encoder. Motion sequences with varying complexity were selected from the RoNIN and iIMU-TD datasets. In the visualizations, dashed lines denote ground-truth trajectories, and solid lines represent predictions from different models. The results are shown in Figure 4.

The results show that in Figure 4a, under low-complexity linear motion, all models produce trajectories close to the ground truth, with LKAN achieving the best alignment. In Figure 4b, as motion complexity increases, ResT-IMU begins to exhibit noticeable deviation, while RoNIN-ResNet and IMUNet show reduced fitting accuracy; in contrast, LKAN maintains high consistency with the ground truth.

In more challenging scenarios, the performance gap becomes more pronounced. In Figure 4c, with frequent turning, the errors of RoNIN-ResNet and IMUNet increase significantly, and ResT-IMU degrades further, whereas LKAN still preserves accurate trajectory alignment. In Figure 4d, corresponding to the highest motion complexity, the trajectories of ResT-IMU, RoNIN-ResNet, and IMUNet exhibit severe drift or dispersion, while LKAN continues to closely follow the ground-truth trajectory.

These results demonstrate that the proposed LKAN framework exhibits clear advantages in modeling strong nonlinear dynamics and maintaining stability under complex motion patterns. Moreover, it achieves consistently robust performance across different datasets and varying motion complexities.

- Verification of Nonlinear Fitting Ability of the KANmer encoder

**Figure 5:**
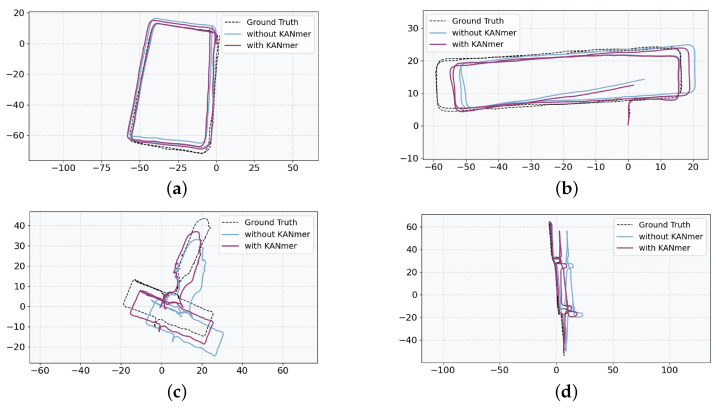
Trajectory comparison results between models with and without the KANmer encoder are illustrated under both simple motion patterns—(**a**) iIMU-TD-11011542a1b1c1 and (**b**) iIMU-TD-11011544a1b1c1—and complex motion patterns—(**c**) RoNIN-a012_2 and (**d**) RoNIN-a001_2. Axes denote spatial coordinates in meters (m).

This experiment evaluated the impact of the KANmer encoder by comparing the trajectory fitting performance of LKAN with and without it (replaced by an MLP) under both simple and complex motion patterns. The results are illustrated by sequences (a) and (b) from iIMU-TD, representing simple motions, and (c) and (d) from RoNIN, representing complex motions. The results are shown in Figure 5.

The results show that in simple motion scenarios (Figure 5a,b), both models achieve comparable trajectory fitting performance, and the advantage of the KANmer encoder remains relatively limited. However, as motion complexity increases (Figure 5c,d), the performance gap becomes significant. The model without KANmer exhibits noticeable trajectory deviation, whereas the full LKAN model maintains accurate alignment with the ground truth.

These results indicate that the KANmer encoder provides enhanced nonlinear representation capability, which becomes particularly critical in modeling complex motion patterns. By effectively capturing high-frequency and strongly nonlinear dynamics in IMU data, it plays a key role in improving trajectory estimation accuracy and robustness under challenging scenarios.

#### 3.4.6. Analysis of the Weight Parameter for the LHSR Module

**Figure 6:**
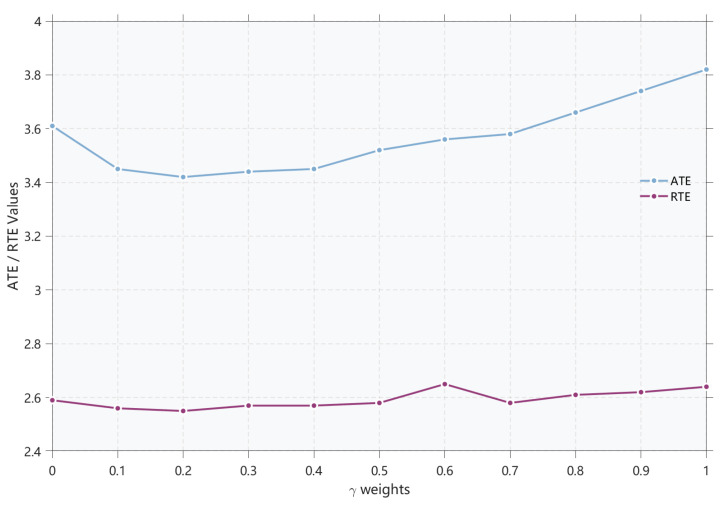
Experimental results of metric comparison on the RoNIN dataset under different γ values. The value of γ is varied from 0.0 to 0.5 with a step of 0.1, while other hyperparameters are kept fixed. The corresponding results are: γ=0.0 (ATE = 3.61, RTE = 2.59), γ=0.1 (ATE = 3.45, RTE = 2.56), γ=0.2 (ATE = 3.42, RTE = 2.55), γ=0.3 (ATE = 3.44, RTE = 2.57), γ=0.4 (ATE = 3.45, RTE = 2.57), and γ=0.5 (ATE = 3.47, RTE = 2.60). When γ>0.5, both ATE and RTE increase, indicating performance degradation.

To investigate the effect of the constraint weight γ (defined in Equation (15)) in the long-range statistical consistency regularization module, a parameter sensitivity analysis was conducted on the RoNIN dataset. Model performance was evaluated using ATE and RTE metrics. The results are shown in Figure 6.

The results show that when γ=0, without the long-range statistical constraint, both ATE and RTE are relatively high, indicating poor global trajectory consistency. As γ increases from 0.0 to 0.2, both metrics decrease steadily and reach their minimum at γ=0.2, where the model achieves optimal performance. When γ further increases beyond 0.2, both ATE and RTE begin to rise, indicating performance degradation. The improvement tends to saturate around γ=0.2, suggesting that the contribution of the regularization term reaches its effective limit and does not provide further gains in global consistency.

This behavior can be attributed to the trade-off introduced by the LHSR constraint, where LHSR serves as an auxiliary regularization term and the primary trajectory estimation capability is provided by the proposed KANmer encoder through nonlinear temporal feature modeling and velocity regression. While a moderate value of γ effectively enhances global consistency by leveraging long-term statistical information, excessively large values weaken the contribution of the main regression objective, leading to degraded local motion modeling and increased ATE and RTE.

Based on these observations, γ=0.2 is selected as the optimal setting, achieving a balanced trade-off between global consistency and local trajectory accuracy.

#### 3.4.7. Error Distribution Analysis of Different Models

**Figure 7:**
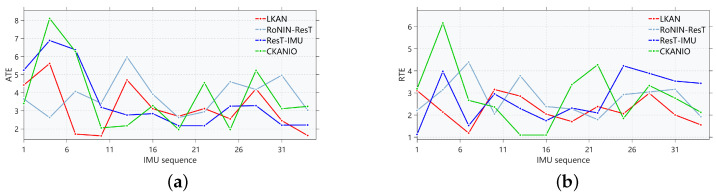
(**a**) Comparison of the average Absolute Trajectory Error of different methods on the RoNIN dataset. (**b**) Comparison of the average Relative Trajectory Error of different methods on the RoNIN dataset. The horizontal axis represents the IMU sequence index, and the vertical axis denotes the ATE/RTE values (in meters). Different colored curves correspond to different methods: red (LKAN), light blue (RoNIN-ResT), dark blue (ResT-IMU), and green (CKANIO).

In this experiment, thirty-five IMU trajectory sequences were randomly selected from the RoNIN dataset. Absolute Trajectory Error and Relative Trajectory Error were adopted as the primary evaluation metrics to compare the performance differences between the proposed LKAN method and three baseline methods, namely RoNIN-ResT, ResT-IMU, and CKANIO. To avoid cluttered visualization caused by plotting all trajectories, the ATE and RTE metrics were averaged over every three consecutive trajectories. The resulting smoothed curves were then used to clearly illustrate the error trends and performance comparisons among different methods. The results are shown in Figure 7.

The results show that the proposed LKAN method achieves the lowest ATE and RTE values across the majority of trajectory segments. Compared with the baseline methods, including RoNIN-ResT, ResT-IMU, and CKANIO, LKAN not only effectively suppresses ATE peaks and fluctuations caused by cumulative IMU drift but also significantly reduces short-term RTE in relative trajectory tracking, without noticeable abrupt variations. Furthermore, LKAN demonstrates superior performance in both long-term absolute positioning accuracy and short-term trajectory robustness, thereby validating its effectiveness and superiority in inertial navigation trajectory estimation tasks.

#### 3.4.8. Analysis of Grid Size *G* and Spline Order *K* in KAN-Based Architecture

**Figure 8:**
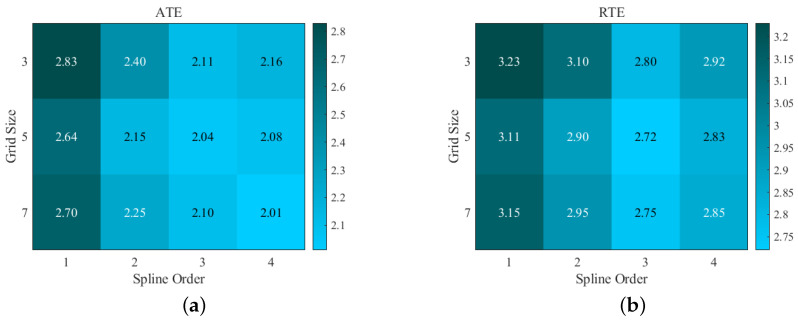
(**a**) Heatmap of ATE under different configurations of grid size *G* and spline order *k* on the iIMU-TD dataset; (**b**) Heatmap of RTE under different configurations of grid size *G* and spline order *k* on the iIMU-TD dataset. Rows correspond to grid size *G*∈{3,5,7}, and columns correspond to spline order *k*∈{1,2,3,4}. Lighter colors indicate lower errors and better trajectory estimation performance.

To further investigate the influence of the structural hyperparameters in the KAN-based encoder, a sensitivity analysis was conducted on the grid size *G* and spline order *k*. Specifically, *G*∈{3,5,7} and *k*∈{1,2,3,4} were evaluated. The experiment was performed on the iIMU-TD dataset, and model performance was measured using ATE and RTE. The results were visualized using heatmaps, as shown in Figure 8.

The experimental results indicate that the performance of the KAN-based encoder is sensitive to the grid size *G* and spline order *k*, and their proper configuration is crucial for stable and accurate trajectory estimation. As shown in the heatmap, the model exhibits a clear non-monotonic trend with respect to *G* and *k*. When both values are small, the model lacks sufficient nonlinear representation capacity to capture the complex dynamics of IMU signals, resulting in higher errors. In contrast, excessively large values increase model complexity and the risk of overfitting, while also introducing additional computational cost, which degrades performance.

Quantitatively, the lowest ATE is achieved at G=7, k=4, with a value of 2.01. However, the configuration of G=5 and k=3 achieves a competitive ATE of 2.04, ranking second overall in terms of ATE, while simultaneously yielding the best RTE of 2.72. Overall, this configuration provides the best trade-off between the two metrics, indicating that a moderate grid size and spline order effectively balance representation capability and generalization while avoiding overfitting.

Therefore, G=5 and k=3 are selected as the default configuration, as they achieve the best balance between accuracy and robustness for IMU-based trajectory estimation tasks.

## 4. Conclusions

To address the challenges of accurately modeling complex nonlinear IMU dynamics and mitigating long-term error accumulation in inertial integration, this paper proposes a novel inertial trajectory estimation framework, termed LKAN, based on the Kolmogorov–Arnold Network (KAN). By constructing a KANmer encoder that integrates KAN with the MHSA mechanism, the proposed framework enables joint modeling of strong nonlinear motion patterns and long-range temporal dependencies in IMU sequences, thereby effectively suppressing trajectory drift and improving global consistency.

Theoretical analysis based on the Kolmogorov–Arnold representation theorem shows that the proposed method decomposes high-dimensional nonlinear mappings of IMU signals into univariate function approximation and linear combination, while learnable B-spline parameterization further enhances the model’s nonlinear fitting capability. From an architectural perspective, MHSA captures long-term temporal dependencies, whereas KAN layers perform fine-grained nonlinear regression on attention-enhanced features. Their synergy allows accurate characterization of complex motion patterns such as gait transitions, acceleration–deceleration, and turning.

Experimental results demonstrate that the proposed LKAN framework achieves superior accuracy and robustness compared with conventional CNN and Transformer-based methods. By effectively balancing nonlinear representation and temporal modeling, the framework significantly reduces error accumulation and improves trajectory stability.

Future work will focus on enhancing the adaptive approximation capability of KAN, exploring its integration with other temporal modeling paradigms, and extending the framework to multi-modal inertial navigation and motion understanding tasks.

## Figures and Tables

**Table 1:** Results of core component ablation study.

KANmer	LHSR (Only Training Phase)	AWM	RoNIN		iIMU-TD	
			ATE (m)	RTE (m)	ATE (m)	RTE (m)
√	√	√	3.42	2.55	2.04	2.72
	√	√	3.82	2.64	2.86	3.53
√		√	3.61	2.59	2.48	3.17
√	√		3.48	2.54	2.14	3.24

**Table 2:** Results of LHSR stage configuration comparison experiment and mechanistic ablation experiment.

	RoNIN		iIMU-TD	
	ATE (m)	RTE (m)	ATE (m)	RTE (m)
**Only Training Phase**	3.42	2.55	2.04	2.72
**Only Inference Phase**	4.23	2.98	2.54	2.77
**Training & Inference Phases**	3.27	2.61	2.06	2.68
**Without LHSR**	3.61	2.59	2.48	3.17
**Disputed LHSR**	3.58	2.67	2.34	2.91

**Table 3:** Experimental results of complexity and efficiency comparison for different model configurations.

	Parameters (M)	Inference Time (ms)	ATE (m)	RTE (m)
**Transformer**	24.22	6.43	3.13	3.76
**KANmer**	24.29	6.74	2.48	3.17
**KANmer + LHSR**	24.34	6.95	2.04	2.72

**Table 4:** Results of comparison experiment.

	iIMU-TD		RoNIN		OXIOD	
	ATE (m)	RTE (m)	ATE (m)	RTE (m)	ATE (m)	RTE (m)
**R-ResNet**	3.27	3.73	3.91	2.76	3.14	2.66
**R-LSTM**	3.87	4.37	4.22	2.73	3.51	2.51
**R-TCN**	3.81	4.92	8.92	8.49	3.33	**1.19**
**ResT-IMU**	3.08	3.95	3.64	2.60	3.23	2.48
**IMUNet**	3.69	3.91	3.96	2.93	**2.88**	2.58
**CKANIO**	4.84	4.03	3.81	3.27	3.62	2.35
**LKAN (ours)**	**2.04**	**2.72**	**3.42**	**2.55**	3.11	2.49

## Data Availability

Data Availability Statement: The data used in this study include both publicly available third-party datasets and self-collected data.The RoNIN and OXIOD datasets are publicly available and can be obtained from their original sources: RoNIN: https://www.frdr-dfdr.ca/repo/dataset/816d1e8c-1fc3-47ff-b8ea-a36ff51d682a (accessed on 15 November 2025); OXIOD: http://deepio.cs.ox.ac.uk/ (accessed on 15 November 2025). These datasets are subject to the terms and conditions specified by their respective providers.The iIMU-TD dataset was collected by our research group. Due to data management considerations, this dataset, along with the source code and raw experimental data, is available from the corresponding author upon reasonable request.
